# Supplementary material for: Introducing Fluorescence-Guided Surgery for Pediatric Ewing, Osteo-, and Rhabdomyosarcomas: A Literature Review
Source: Biomedicines. 2021 Oct 4;9(10):1388. doi: 10.3390/biomedicines9101388 (PMC8533294; doi:10.3390/biomedicines9101388)
Supplement: Supplementary file 1 [file biomedicines-09-01388-s001.zip › biomedicines-1331889-supplementary.pdf]

Table S1. Published/completed trials with clinically available antibodies for Osteosarcoma-, Rhabdomyosarcoma- and Ewing Sarcoma patients

Targets	Tumor type	Clinically available antibody (targeted therapy)	Age of the study population	Clinical trial phase	Safety	Literature (PMID/NCT)
HER2	Metastatic OS	Trastuzumab	<32-year-old patients	Phase 2	Safe	22665540
gpNMB	Recurrent OS	Glembatumumab vedotin	12–31-year-old patients	Phase 2	Safe	31586757
VEGF-A	OS, ES, and RMS	Bevacizumab	0.5-29-year-old patients	Pilot- and phase 2	Safe (one study reported wound complications)	28631382, 26461056, 23630159, 31513481, 28738258
IGF-1R	OS, ES, and RMS	Cixutumumab, Ganitumab, Teprotumumab, Figitumumab, Robatumumab and Dalotuzumab	1–85-year-old patients	Pilot-, phase 1 and phase 2	Safe	30351457, 22508822, 22025149, 20371689, 22465830, 25446280, 23956055, 22184397, 23477833, 22025154, 27362300, 24797726, 21127194, 23835252, 21750201, 27185573, 20036194
TRAIL-R2	Recurrent or progressive OS, ES, and RMS	Lexatumumab	2–21-year-old patients	Phase 1	Safe	23071222
TEM1	OS, recurrent or refractory ES and RMS	Ontuxizumab	3–21-year-old patients	Phase 1	Safe	29292843

B7-H3	Relapsed/refractory OS, ES, and RMS	Enoblituzumab	1–35-year-old patients	Phase 1	No statement on safety	NCT02982941

Table S2. Ongoing/unpublished trials with clinically available antibodies for Osteosarcoma-, Rhabdomyosarcoma- and Ewing Sarcoma patients

Targets	Tumor type	Clinically available antibody (targeted therapy)	Age of the study population	Clinical trial phase	Status	NCT-number
GD2	OS	Hu3F8-BsAb (humanized 3F8 bispecific antibody) and Dinutuximab	1–40-year-old patients	Phase 1 and phase 2	Recruiting<br>Active, not recruiting<br>Not yet recruiting	NCT02502786 and NCT03860207<br>NCT02484443<br>NCT04751383
HER2	OS	Trastuzumab Deruxtecan (ADC)	12 – 39-year-old patients	Phase 2	Recruiting	NCT04616560
PD-L1	OS	ZKAB001, Avelumab and Durvalimub	12–55-year-old patients	Phase 1, phase 2 and phase 3	Recruiting<br>Active, not recruiting<br>Not yet recruiting	NCT03676985 and NCT04668300<br>NCT03006848<br>NCT04359550
CD47 and GD2	OS	Magrolimab + Dinutuximab	1–35-year-old patients	1	Not yet recruiting	NCT04751383
semaphorin 4D	Recurrent or refractory OS	Pepinemab	1–30-year-old patients	1-2	Active, not recruiting	NCT03320330

AXL	OS and ES	CAB-ANTI-AXL-ADC	> 17-year-old patients	1-2	Recruiting	NCT03425279
IGF-1R	ES, Embryonal and Alveolar RMS	Ganitumab	> 1-year-old patients<br>< 51-year-old patients	Phase 1, phase 2 and phase 3	Recruiting<br>Active, not recruiting	NCT03041701<br>NCT02306161
CD56	RMS	Lorvotuzumab Mertansin (ADC)	1–30-year-old patients	2	Active, not recruiting	NCT02452554
